# Circulating IL-17A Levels in Postmenopausal Women with Primary Hyperparathyroidism

**DOI:** 10.1155/2020/3417329

**Published:** 2020-01-17

**Authors:** E. Dozio, E. Passeri, E. Vianello, S. Palmieri, C. Eller-Vainicher, M. Corsi Romanelli, S. Corbetta

**Affiliations:** ^1^Department of Biomedical Sciences for Health, University of Milan, Milan, Italy; ^2^Endocrinology and Diabetology Service, IRCCS Istituto Ortopedico Galeazzi, Milan, Italy; ^3^Endocrine Unit, IRCCS Fondazione Cà Granda Ospedale Maggiore Policlinico, Milan, Italy; ^4^UOC SMEL-1, IRCCS Policlinico San Donato, San Donato Milanese, Milan, Italy; ^5^Department of Biomedical, Surgical and Dental Sciences, University of Milan, Milan, Italy

## Abstract

**Background:**

Primary hyperparathyroidism (PHPT) is a common cause of secondary osteoporosis in postmenopausal women. Th17 lymphocytes and the released cytokine IL-17A play an important role in bone metabolism. Th17 cells have been shown to be activated by PTH, and peripheral blood T cells from patients affected with PHPT express higher levels of IL-17A mRNA than controls.

**Aim:**

To investigate circulating levels of IL-17A and the ratio RANKL/OPG, as markers of osteoclastogenesis, in 50 postmenopausal PHPT women compared with postmenopausal osteoporotic non-PHPT women (*n* = 20).

**Results:**

Circulating levels of IL-17A were similarly detectable in most PHPT and non-PHPT osteoporotic women (12.9 (8.4-23.1) vs. 11.3 (8.3-14.3) pg/ml, median (range interquartile), *P* = 0.759), at variance with premenopausal women where IL-17A was undetectable. In PHPT women, any significant correlations could be detected between circulating IL-17A levels and PTH levels. Nonetheless, significant negative correlations between circulating IL-17A and ionized calcium levels (*r* = ‐0.294, *P* = 0.047) and urine calcium excretions (*r* = ‐0.300, *P* = 0.045) were found. Moreover, PHPT women were characterized by positive correlations between IL-17A levels and femur neck (*r* = 0.364, *P* = 0.021) and total hip (*r* = 0.353, *P* = 0.015) *T*-scores. Circulating IL-17A levels did not show any significant correlation with sRANKL, OPG, and sRANKL/OPG ratio in PHPT women.

**Conclusions:**

In postmenopausal PHPT women, circulating IL-17A levels were similar to those detected in postmenopausal non-PHPT women, showing a disruption of the relationship observed in postmenopausal osteoporosis among circulating PTH, sRANKL, OPG, IL-17A, and bone demineralization in postmenopausal PHPT women. The data support an osteogenic effect of IL-17A in postmenopausal PHPT women.

## 1. Introduction

Primary hyperparathyroidism (PHPT) is a common cause of secondary osteoporosis in postmenopausal women [1]. Complex integration between immune system and skeleton is emerging. T cells, by controlling B cell osteoprotegerin (OPG) production and hence the balance of active key osteoclastogenic cytokine receptor activator of NF-*κ*B ligand (RANKL), regulate basal osteoclastogenesis and bone resorption [2]. Disruption of the interaction of the immune-skeletal system interface is recognized to play an important role in the common estrogen deficiency-related postmenopausal osteoporosis [3] and also in PHPT-related osteoporosis [4]. PTH exerts its effects on bone by activating the PTH/PTH-related peptide (PTHrp) receptor PTHR1 of bone marrow stromal cells, osteoblasts, and osteocytes and also of T cells [5]. In mice, continuous PTH stimulates T cell production of TNF*α*, which increases the differentiation of IL-17A-producing Th17 cells via TNF receptor 1 (TNFR1) signaling in CD4^+^ cells [6]. In PHPT patients, where PTH release is inappropriately and persistently elevated, Li et al. reported that peripheral blood cell expression of *IL-17A* mRNA was increased and normalized after successful parathyroidectomy [6]. The authors speculated that neutralization of IL-17A might represent a novel therapeutic strategy for PHPT-related osteoporosis; this perspective is fascinating as an IL-17A inhibitor, secukinumab, has been developed for the treatment of ankylosing spondylitis [7]. IL-17A, also known as IL-17, is produced by the bone marrow Th17 cells, which are an osteoclastogenic population of CD4^+^ cells [8]. IL-17A stimulates the release of RANKL by all osteoblastic cells including osteocytes [9, 10] and potentiates the osteoclastogenic activity of RANKL by upregulating RANK [11]. PTH has been recently reported to indirectly increase osteocytic RANKL expression, through an IL-17A/IL-17A receptor-mediated mechanism [10]. The interaction between RANK and RANKL is further modulated by a soluble “decoy” receptor called OPG, which also binds to RANK but does not induce osteoclastogenesis [12]. The relative balance between OPG and RANKL dictates the magnitude of osteoclastogenesis.

Here, we evaluated circulating levels of IL-17A and of the ratio RANKL/OPG in postmenopausal PHPT patients compared with postmenopausal osteoporotic non-PHPT women. We showed that serum IL-17A levels in PHPT postmenopausal women (1) were similar to those detected in normocalcemic non-PHPT postmenopausal women, (2) negatively correlated with biochemical parameters of PHPT, and (3) correlated with the bone mineral density status.

## 2. Patients and Methods

### 2.1. Patients

Fifty female postmenopausal patients with diagnosis of PHPT (elevated serum albumin-corrected and/or ionized calcium and inappropriately elevated serum PTH levels) were consecutively enrolled in the third level academic centers of IRCCS Istituto Ortopedico Galeazzi and IRCCS Fondazione Cà Granda Ospedale Maggiore Policlinico in Milan. Twenty aged-matched female postmenopausal healthy women were enrolled as controls. Moreover, further 45 healthy normocalcemic premenopausal women without diagnosis of osteoporosis were evaluated as controls. Clinical and biochemical features are shown in Table 1 and Supplementary [Supplementary-material supplementary-material-1]. IL-17A is known to play a role in autoimmune and inflammatory diseases, including rheumatoid arthritis, psoriasis, multiple sclerosis, asthma, and inflammatory bowel disease [13, 14]. Therefore, PHPT patients and controls were investigated by extensive clinical and biochemical evaluations, and subjects with history and/or biochemical markers of autoimmune diseases, including Hashimoto's thyroiditis, nephropathy, active cancer, or hematopathy, were excluded from the enrollment. Active smokers and drinkers were also excluded. All PHPT patients and controls were evaluated in the absence of drugs known to affect bone metabolism, in particular glucocorticoids, thiazide diuretics, calcium and vitamin [Supplementary-material supplementary-material-1]ementation, bisphosphonates, denosumab, and teriparatide. Most of the PHPT patients (80%) were supplemented with cholecalciferol.

This study was carried out in accordance with the recommendations of IRCCS Ospedale San Raffaele Milan Ethical Committee with written informed consent from all subjects. All subjects gave written informed consent in accordance with the Declaration of Helsinki. The protocol was approved by the local Ethical Committee.

### 2.2. Clinical, Biochemical, and Hormonal Assessment

PHPT patients were investigated by dual-energy X-ray absorptiometry (DEXA) scanner for the measurement of segmental bone mineral density (BMD) using a Hologic densitometer. Participants were scanned in light clothing, while lying flat on their backs with arms at their sides.

Venous blood samples were collected after an overnight fasting in all patients. Serum calcium, ionized calcium, phosphate, creatinine, and total alkaline phosphatase were measured according to the routinely used laboratory kits. Serum PTH levels were determined by electrochemiluminescence on an Elecsys 2010 (Roche Diagnostics, Mannheim, Germany), and serum 25-hydroxyvitamin D (25OHD) was measured by a chemiluminescent assay (LIAISON® test, DiaSorin Inc., Stillwater, MN, USA). Plasma IL-17A levels were assayed by Human IL-17 Quantikine ELISA kit (R&D System, Cat. D1700; intra-assay CV 4.7%, interassay CV 8.4%, sensitivity <3.0 pg/ml, by considering an additional point to the low range of the standard curve); plasma OPG was determined by the Human Osteoprotegerin ELISA kit (BioVendor, Cat. RD194003200; intra-assay CV 4.9%, interassay CV 9.0%, sensitivity 0.03 pmol/l) and plasma soluble RANKL by the sRANKL (total) human ELISA kit (BioVendor, Cat. RD193004200R; intra-assay CV 11.5%, inter-assay CV 12.7%, sensitivity 0.4 pmol/l).

### 2.3. Statistical Analysis

Normally distributed data are expressed as mean ± SD, while data failing to pass the D'Agostino-Pearson normality test are expressed as median and range interquartile. Groups were compared using *T*-test for normal variables or Mann–Whitney *U*-test for nonparametric variables. Simple correlation analyses were performed by using the Pearson or Spearman correlation as indicated. A *P* value less than 0.05 was considered significant. Statistical analysis was performed using GraphPad Prism® 6.0c. Considering as a significant difference of at least 30% between the mean IL-17A levels detected in postmenopausal PHPT women compared with the mean levels detected in postmenopausal controls, a sample size of 50 patients provided the ability to detect a statistically different incidence of the variation between the two groups with a power of 0.80 and an alpha of 0.05.

## 3. Results

### 3.1. Circulating IL-17A Levels in Postmenopausal PHPT Women

**Figure 1:**
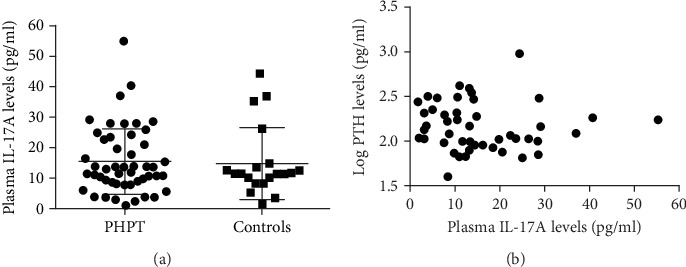
(a) Circulating IL-17A levels in postmenopausal PHPT women (black circles) compared with postmenopausal non-PHPT women (controls; black squares). (b) Correlation between circulating IL-17A levels and log PTH levels in PHPT women.

Circulating levels of IL-17A were undetectable in all samples from the series of premenopausal women, similar to what previously reported [15, 16]. At variance, plasma levels of IL-17A were measurable in most PHPT and non-PHPT osteoporotic postmenopausal women. PHPT and non-PHPT osteoporotic women showed similar circulating IL-17A levels (Figure 1(a)), and no significant correlation with age could be detected (*P* = 0.261). Median sRANKL and OPG levels did not differ between the two groups (Table 1).

**Figure 2:**
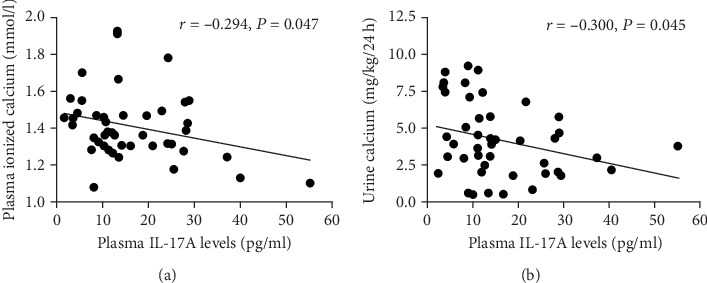
(a) Correlation between circulating IL-17A and plasma ionized calcium levels in PHPT women. (b) Correlation between circulating IL-17A and urine calcium excretion levels in PHPT women.

In PHPT women, any significant correlations could be detected between circulating IL-17A levels and PTH levels (Figure 1(b)), while significant negative correlations among circulating IL-17A and ionized calcium levels (*r* = ‐0.294, *P* = 0.047) (Figure 2(a)) and body weight-corrected urine calcium excretions (*r* = ‐0.300, *P* = 0.045) (Figure 2(b)) were found. Soluble RANKL did not correlate with any of the biochemical markers of PHPT disease, except for 25OHD levels (*r* = 0.334, *P* = 0.029) (Supplemental [Supplementary-material supplementary-material-1], panel A), while circulating OPG levels positively correlated with PTH levels (*r* = 0.350, *P* = 0.016) (Supplemental [Supplementary-material supplementary-material-1], panel B).

### 3.2. Correlations between Circulating IL-17A and Bone Mineral Density in Postmenopausal PHPT Women

**Figure 3:**
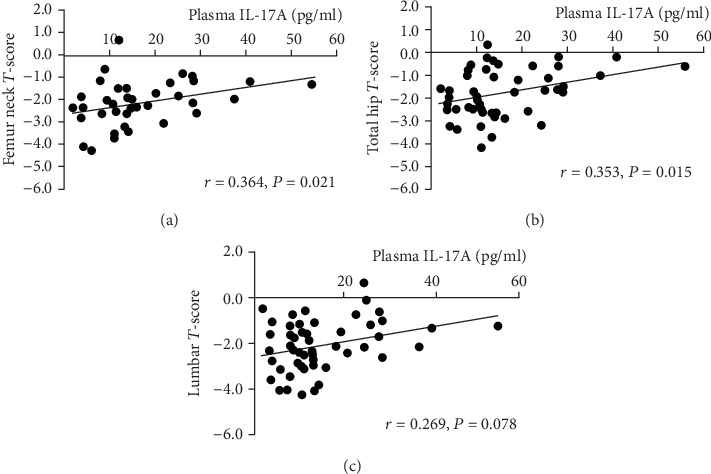
(a) Correlation between circulating IL-17A and femur neck *T*-scores in PHPT women. (b) Correlation between circulating IL-17A and total hip *T*-scores in PHPT women. (c) Correlation between circulating IL-17A and lumbar *T*-scores in PHPT women.

PHPT women were characterized by significant and positive correlations between IL-17A levels and femur neck (*r* = 0.364, *P* = 0.021) (Figure 3(a)) and total hip (*r* = 0.353, *P* = 0.015) (Figure 3(b)) *T*-scores. Indeed, a positive correlation between IL-17A levels and lumbar spine *T*-score (*r* = 0.269, *P* = 0.078) (Figure 3(c)) was observed, though it failed to get the statistical significance.

### 3.3. Correlations between Circulating IL-17A and Bone Markers in Postmenopausal PHPT Women

In PHPT women, circulating IL-17A levels did not show any significant correlation with sRANKL, OPG, and sRANKL/OPG ratio. Similarly, IL-17A did not correlate with total alkaline phosphatase activity levels. At variance, in non-PHPT osteoporotic women, IL-17A levels positively correlated with OPG levels (*r* = 0.681, *P* = 0.004).

## 4. Discussion

Continuous PTH induces Th17 cell differentiation in the bone marrow and IL-17A produced by bone marrow Th17 cells may act as upstream cytokine that plays a pivotal role in the bone loss induced by continuous PTH and PHPT. IL-17A, released by a T cell subset, mediates potent osteoclastogenic activity by stimulating the production of RANKL from osteoblast progenitors. T lymphocytes express the functional PTH receptor PTH-R1, respond to PTH, and stimulate osteoblast differentiation [5]. In the study by Li et al., PTH levels were directly correlated with lymphocytic mRNA levels of *IL-17A* and increased *IL-17A* gene expression in PHPT patients was likely due to increased levels of circulating PTH [6]. Whether PHPT increases levels of IL-17A protein in the peripheral circulation and in the bone marrow remains to be determined.

Here, we firstly investigated the circulating levels of IL-17A in a well selected and characterized series of postmenopausal women affected with PHPT. Both PHPT and non-PHPT patients affected with autoimmune or inflammatory diseases were carefully excluded; therefore, circulating IL-17A can be assumed deriving from bone marrow leakage. When compared with a series of postmenopausal non-PHPT osteoporotic women, PHPT women showed similar circulating IL-17A levels. By contrast, circulating IL-17A was undetectable in premenopausal women without osteoporosis, in line with previous reports [15, 16], and consistent with the inhibitory effect of estrogens on IL-17A release. Therefore, postmenopausal estrogen withdrawal may be one of the determinants of the rise in plasma IL-17A levels, which was detectable in most of PHPT and non-PHPT postmenopausal women.

In the present series of PHPT patients, mean levels of circulating IL-17A, likely derived from bone resident lymphocytes and osteoblasts [17, 18], were higher in PHPT women with mild hypercalcemia and calciuria with respect to PHPT women with more severe hypercalcemia and hypercalciuria. Similarly, IL-17A levels were higher in PHPT women with osteopenia with respect to PHPT women with osteoporosis. Indeed, our findings were in contrast with the data by Li et al. [6], who reported a positive relationship between peripheral blood lymphocytic *IL-17A* transcripts and serum PTH levels in PHPT patients, and with the finding in postmenopausal osteoporotic women, where circulating IL-17A levels negatively correlate with the lumbar bone mineral density (BMD) [19, 20]. It is difficult to provide an explanation for this discrepancy. However, it should be considered that the expression levels of *IL-17A* transcripts in lymphocytes from peripheral blood, investigated by Li et al. [6], may not invariably correlate with the IL-17A protein levels in the plasma of PHPT patients, likely deriving from the bone resident lymphocytes and osteoblasts, due to posttranscriptional modulation. Immune system seems to play an important role in bone remodeling through the secretion of many cytokines that can exert different functions, such as influencing osteoclast differentiation and activation (*i.e.*, IL-6, TNF*α*, IFN*γ*), protecting against bone resorption (*i.e.*, IL-4, IL-12, IL-33), modulating T cell phenotype, and the secretion of other cytokines, chemokines, and metalloproteases [21]. Therapeutic options capable to reducing the risk of bone loss by controlling inflammation are emerging approaches to osteoporosis management [22]. However, the crosstalk between the immune system and bone is not so clear-cut as well as the role of many mediators, which could be strongly influenced by the variable cytokine network expressed in a specific clinical condition. In fact, there are conflicting results regarding the positive/negative effects of inflammatory/anti-inflammatory cytokines, including IL-17A, on osteoblast differentiation [23]. It has been reported that IL-17A induces osteogenesis in physiological conditions, while excessive IL-17A production, as the experimental condition in continuous PTH-treated mice, results in enhanced RANKL secretion and osteoclastogenesis [24, 25]. Therefore, we are tempting to speculate that the detectable IL-17A levels in the plasma of the PHPT women, well below the levels measured in patients with inflammatory diseases, might reflect the IL-17A osteogenic effect, consistent with the positive correlations with the *T*-scores and the negative correlations with the markers of PHPT severity, rather than the proosteoclastogenic effect [11]. Moreover, the present study failed in detecting any correlation between IL-17A and bone markers of osteoclast activity, such as sRANKL and OPG, which were similar to those detected in non-PHPT women.

It should be considered that most of the data about the interaction among PTH, Th17 lymphocytes, IL-17A, and RANKL have been accumulated investigating murine models of continuous PTH infusion. Though a recent report demonstrated that in mice, continuous PTH augments RANKL expression indirectly, via an IL-17A/IL-17A receptor-mediated mechanism [10], Kimura et al. investigating sRANKL in PHPT patients failed to detect any correlation between sRANKL and PTH levels [25]. Therefore, the lack of correlation in the present series of PHPT women between serum PTH levels and circulating sRANKL as well as IL-17A levels, considered as markers of osteoclastogenesis, seems to be in line with the finding by Nackchbandi et al. [26] and supports the osteogenic effect of IL-17A in postmenopausal PHPT women. Of note, in the present PHPT series, OPG, the RANKL inhibitor that inhibits osteoclastogenesis, positively correlated with PTH.

Admittedly, the present data suffer from limitations, first of all, the cross-sectional design of the study and the small size of the analyzed series, though PHPT patients enrolled were extensively screened to exclude any disease known to impair IL-17A release from T cells. Secondly, we could not analyze the IL-17A levels in bone marrow biopsies from postmenopausal PHPT patients in order to confirm the bone marrow leakage derivation of the circulating IL-17A.

## 5. Conclusions

Circulating IL-17A levels in postmenopausal PHPT women were similar to those detected in postmenopausal non-PHPT women, though they likely reflect the IL-17A osteogenic rather than the proosteoclastic effect.

## Figures and Tables

**Table 1:** Clinical and biochemical parameters in postmenopausal PHPT women and controls.

Parameters	nv	PHPT	Controls	*P*
*n*	—	50	20	—
Anthropometric parameters				
Age (years)	—	66.5 (58.8-74.0)	69.0 (57.0-75.0)	0.465
BMI (kg/m^2^)	—	25.6 (22.9-28.7)	25.1 (23.0-28.6)	0.821
Biochemical parameters				
Serum calcium (mg/dl)	8.4-10.4	10.8 (10.4-11.4)	9.5 (9.2-9.8)	**<0.0001**
Ionized calcium (mmol/l)	1.15-1.29	1.40 (1.31-1.48)	—	—
Serum phosphate (mg/dl)	3.5-5.0	2.6 (2.2-2.9)	3.4 (3.2-3.8)	**<0.0001**
Serum PTH (pg/ml)	10.0-65.0	135.5 (93.7-212.8)	50.0 (38.0-60.0)	**<0.0001**
Alkaline phosphatase (U/l)	40-120	85.0 (65.5-122.5)	65.0 (57.0-83.0)	**0.003**
25OHD (ng/ml)	30.0-50.0	17.8 (9.4-25.1)	23.7 (17.4-31.8)	**0.005**
eGFR (ml/min)	>60.0	85.0 (65.0-107.5)	79.2 (62.0-102.5)	0.453
Urine calcium (mg/kg/24 h)	<4.0	4.0 (2.2-5.9)	2.3 (1.6-3.3)	**0.020**
sRANKL (pmol/l)	—	105.7 (71.9-163.3)	155.4 (80.9-244.2)	0.148
OPG (pmol/l)	—	6.7 (5.9-8.3)	7.5 (5.7-11.5)	0.117
sRANKL/OPG	—	14.6 (9.7-27.6)	17.9 (10.8-33.1)	0.605
IL17 (pg/ml)	—	13.0 (8.4-23.1)	11.2 (9.6-22.9)	0.787
Bone densitometric parameters				
Lumbar *T*-score	—	−2.15 ± 1.07	−2.91 ± 1.34	**0.028**
Femur neck *T*-score	—	‐2.15 ± 0.98	‐2.45 ± 0.92	0.317
Total hip *T*-score	—	‐1.76 ± 1.07	‐2.2 ± 0.91	0.169

PHPT: primary hyperparathyroidism; BMI: body mass index; 25OHD: 25-hydroxyvitamin D; sRANKL: soluble receptor activator of NF-*κ*B ligand; OPG: osteoprotegerin; IL17: interleukin 17.

## Data Availability

The datasets generated during and/or analyzed during the current study are available from the corresponding author on reasonable request.

## Supplementary Materials

Supplementary Materials Supplementary Figure 1: (A) correlation between serum sRANKL and 25OHD levels in PHPT patients and (B) correlation between serum OPG and PTH levels in PHPT patients. Supplementary Table 1: clinical and biochemical parameters in premenopausal controls. Supplementary Table 2: clinical features of PHPT women.
