# Supplementary material for: Circulating IL-17A Levels in Postmenopausal Women with Primary Hyperparathyroidism
Source: Mediators Inflamm. 2020 Jan 17;2020:3417329. doi: 10.1155/2020/3417329 (PMC7099202; doi:10.1155/2020/3417329)
Supplement: Supplementary Materials — Supplementary Figure 1: (A) correlation between serum sRANKL and 25OHD levels in PHPT patients and (B) correlation between serum OPG and PTH levels in PHPT patients. Supplementary Table 1: clinical and biochemical parameters in premenopausal controls. Supplementary Table 2: clinical features of PHPT women. [file 3417329.f1.pdf]

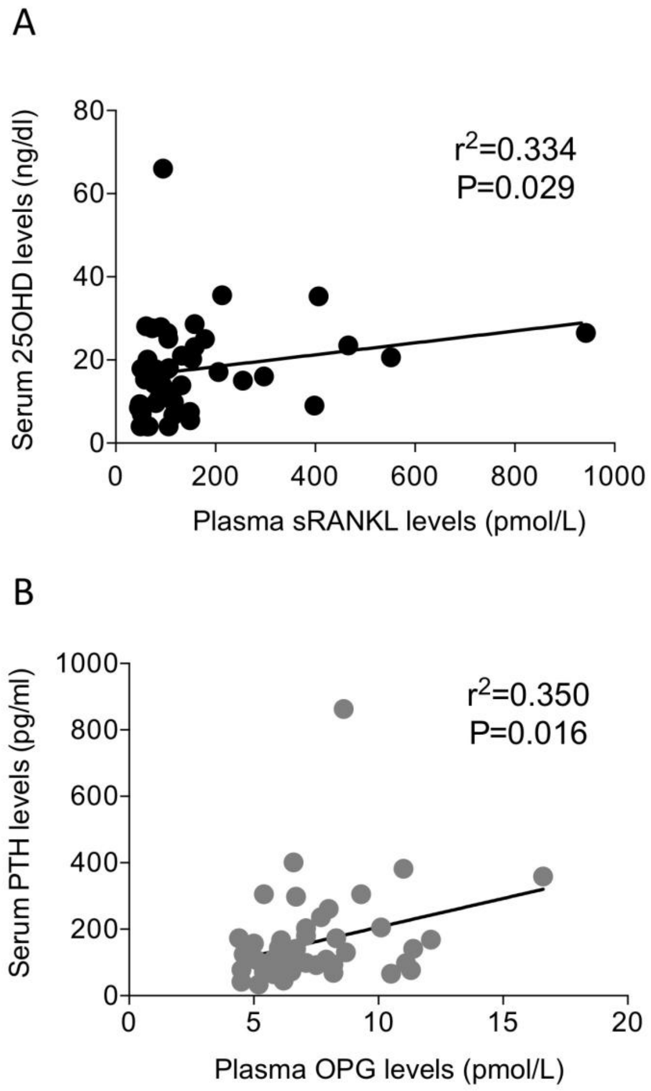

Supplementary Figure 1

Supplementary Table 2. Clinical features of PHPT women.

Features	PHPT patients
N	50
PHPT-related morbidities	
Arterial blood hypertension, %	64.0
Bone fragility fractures, %	18.5
Kidney stones, %	46.3
Other co-morbidities	
Diabetes, %	9.3
Dyslipidemia, %	46.3
Previous cured neoplasia, %	9.3
Non-autoimmune thyroid diseases, %	18.5

Supplementary Table 1. Clinical and biochemical parameters in premenopausal controls.

Parameters	nv	Premenopausal controls
n	-	45
Anthropometric parameters		
Age (years)	-	33.0 (28.0-38.0)
BMI (kg/m^2)	-	28.3 (22.9-33.4)
Biochemical parameters		
Serum calcium* (mg/dl)	8.4-10.4	8.8 (8.6-9.0)
Serum phosphate (mg/dl)	3.5-5.0	3.4 (3.1-3.8)
Serum PTH (pg/ml)	10.0-65.0	51.0 (37.5-66.5)
25OHvitamin D (ng/ml)	30.0-50.00	18.0 (10.1-24.1)
IL-17A (pg/ml)	-	Undetectable
Bone densitometric parameters		
Lumbar Z-score	-	-0.23±1.09
Femur neck Z-score	-	0.03±1.24
Total hip Z-score	-	0.15±1.14

BMI, body mass index; 25OHvitamin D, 25-hydroxyvitamin D; IL1-7A, interleukin 17A.

* Serum albumin-corrected calcium.
